# QAnalysis: a question-answer driven analytic tool on knowledge graphs for leveraging electronic medical records for clinical research

**DOI:** 10.1186/s12911-019-0798-8

**Published:** 2019-04-01

**Authors:** Tong Ruan, Yueqi Huang, Xuli Liu, Yuhang Xia, Ju Gao

**Affiliations:** 1 Department of Computer Science and Engineering, East China University of Science and Technology, Shanghai, 200237 China; 2 Shanghai Shuguang Hospital, Shanghai, 200021 China

**Keywords:** Electronic medical record, Statistical question answering, Graph database, Context-free grammar

## Abstract

**Background:**

While doctors should analyze a large amount of electronic medical record (EMR) data to conduct clinical research, the analyzing process requires information technology (IT) skills, which is difficult for most doctors in China.

**Methods:**

In this paper, we build a novel tool QAnalysis, where doctors enter their analytic requirements in their natural language and then the tool returns charts and tables to the doctors. For a given question from a user, we first segment the sentence, and then we use grammar parser to analyze the structure of the sentence. After linking the segmentations to concepts and predicates in knowledge graphs, we convert the question into a set of triples connected with different kinds of operators. These triples are converted to queries in Cypher, the query language for Neo4j. Finally, the query is executed on Neo4j, and the results shown in terms of tables and charts are returned to the user.

**Results:**

The tool supports top 50 questions we gathered from two hospital departments with the Delphi method. We also gathered 161 questions from clinical research papers with statistical requirements on EMR data. Experimental results show that our tool can directly cover 78.20% of these statistical questions and the precision is as high as 96.36%. Such extension is easy to achieve with the help of knowledge-graph technology we have adopted. The recorded demo can be accessed from https://github.com/NLP-BigDataLab/QAnalysis-project.

**Conclusion:**

Our tool shows great flexibility in processing different kinds of statistic questions, which provides a convenient way for doctors to get statistical results directly in natural language.

## Background

A large amount of EMR data has been accumulated since the wide adoption of medical information tools in China. More hospitals have integrated data from different information tools into clinical data repository (CDR), and regional platforms have also gathered EMR from dozens of hospitals in an area. The data can be utilized in many different types of clinical research. For example, the CALIBER (https://www.ucl.ac.uk/health-informatics/caliber) project, which contains United Kingdom’s EMR data has supported more than ten research projects, including etiology, quality of care and pharmacy.

To conduct clinical research, data queries and analysis on EMR data are not only necessary, but also subject to change according to research topics. However, the research processes are led by doctors who lack IT skills and can hardly write structured query language (SQL) themselves. In this paper, we design a novel tool QAnalysis. The tool allows doctors to enter their analytic requirements with questions in their natural language, and it returns the query results with charts and tables.

While there are many studies on knowledge-based QA [1, 2], our work distinguishes itself from these studies in the following aspects:

1. Questions must be analytics instead of fact-oriented. Existing work focuses on fact-oriented questions. The question always like “Who is Yao Ming’s wife” which can be constructed as a corresponding triple, and the answer is an entity in the knowledge base. Our questions are analytic, and they often include statistical operators, such as ratio, maximum, and average. Besides, a question usually contains logic operators, such as NOT, AND, and OR. Negations and combinations are important concepts in clinical medicine. In general, the answer of a fact-oriented question can be directly extracted from data in the knowledge base. For analytic questions, the answers are derived statistically from data in the knowledge base. In particular, analytic questions may include vocabularies referring to logic operator, and statistic operators such as average and summarization.

2. Questions contain medical terms and require domain knowledge. The questions inevitably have medical terms, such as names of diseases, tests and drugs. These named entities lead to difficulties in parsing questions. Specially, the word-segmentation step may split the word incorrectly. Furthermore, doctors may use types of drugs instead of drug names in the questions. For example, they may ask “The number of patients who use ACEI (angiotension converting enzyme inhibitors) drugs”. However, the EMRs only contain common drug names; therefore, drug knowledge bases may be used to answer these kinds of questions.

3. High accuracy is required. For QA on the common domain, the precision can be fairly low. For example, the best precision score of question answering over linked data (QALD) in 2016 [3] is only 0.61. However, it is not acceptable in our contexts.

The following were done to overcome the aforementioned challenges:

1. We design a graph-based schema about the patients, which can be extended or revised according to different application contexts. The strong schema also provides the basis for the accuracy of question interpretation
2. We use the Delphi method to collect sets of analytic questions from doctors. We design a tree-like knowledge representation to represent possible questions. The representation includes different kinds of operators used in clinical research.
3. We use an existing clinical terminology graph in Chinese [4] in the semantic parsing step to deal with parse issues. Moreover, the patient graph can be linked with the clinical terminology graph (including the drug graph) to support shallow inference-like capabilities.
4. We also present a context-free grammar to guarantee the precision of the tool. Furthermore, the schema of the patient graphs is used when ambiguity exists. As to questions which do not follow the context-free grammar, we use the dependency parsing to improve the coverage of the tool.

The tool consists of three parts: a. Patient data linked with clinical terminologies are represented as knowledge graphs, which provide obvious semantics for relations between medical concepts. b. A parser which transform queries from natural language to Cypher, the query language for Neo4j. c. The Cypher queries are executed on patient data stored in Neo4j. For a given question from a user, the tool first segments the sentence, and then we use grammar parser to analyze the structure of the sentences. After linking the segmentations to concepts and predicates in knowledge bases, we convert the questions into a set of triples connected with different kinds of operators. These triples are converted to queries in Cypher. Finally, the query is executed on Neo4j, and the results shown in tables and charts are returned to the users. The demo can be accessed from GitHub (https://github.com/NLP-BigDataLab/QAnalysis-project). To the best of our knowledge, we design the first QA tool that aims at adhoc clinical analysis purpose. We do not find any similar tool in other domains either.

## Methods

### Overview of the method

We prepare all the data required as knowledge graphs. Specially, we need to build our patient graph G which contains patient-data graph Gd and patient-schema graph Gs. We also link the patient graph with clinical terminology graph. The details are defined in next section. The original EMRs are stored in relational database. We have to export all related data to comma-separated values (CSV) format and import them to Neo4j. Because query parsing needs dictionary and schema information, we extract a dictionary and a concept lexicon from the clinical terminology graph and the patient-schema graph. The former, we have built in the former research work, and the latter, we constructed from the dataset.

**Fig. 1:**
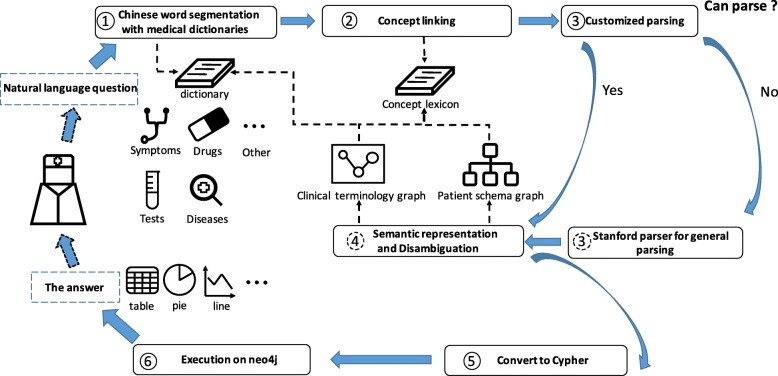
The workflow of our tool

Figure 1 shows the overall work flow of our tool. The process starts from a user submitting a natural-language question to the user interface of the tool. First, the question is segmented into a bag of words with the Chinese word-segmentation tool Jieba (https://github.com/fxsjy/jieba). Since the questions are composed of medical terms, we also add a set of medical dictionaries that include diseases, tests, symptoms and so on to the segmentation tool to improve the accuracy of word segmentation. Jieba can give several possible segmentation results. For example, the original question is “患有高血糖没有患有高血压的病人吃了降血糖类药后葡萄糖化验结果偏低的女患者数量?” (“How many female patients with hyperglycaemia and no hypertension who have low levels of glucose tests after they took hypoglycemic drugs?”). The segmentation result of “女患者” (“female patients”) can be “女/患者” (“female/patients”) or “女患者” (“female patients”). We pass all the possible results to later phase for disambiguation.

Second, we annotate all segments with different concept types. We collect 14 concept types, which consist of Class, Instance, Property, Number, StatisticsOperator, TimeOperator, LogicalOperator (and/or), and so on. For example, TimeOperator connects two time-related events or embeds a TimeValue to represent time. RangeSeparator is used as a delimiter between two numbers, indicating a numeric interval. We use our knowledge base and pattern libraries to annotate different segmentations. Take the previous question as an example. “hypertension” is annotated as an Instance, and “patients” is a Class. The resulting sequence of concept types is “Relation Instance Not Relation Instance Class Relation Instance TimeOperator Instance Property EnumValue Class StatisticsOperator.”

Third, we use a context-free grammar to connect all segments. The context-free grammar provides the normal forms our QA tool supports. The grammar gives hints on the possible triples, systems of logic, and time relations between triples, and the statistical operations on datasets. The original questions may be reduced to different parse trees, and we still delay the disambiguation step to a later phase. If our grammar parser fails to parse the segments, we will call the Stanford parser (https://nlp.stanford.edu/software/lex-parser.shtml) to obtain the dependency tree, and use special rules to convert the user questions into standard forms.

Fourth, the parse tree composed of chunks is transformed into a tree-like internal knowledge representation, which consists of triples connected with various of operators. Multiple candidates may be generated during the process. We use a patient-schema graph to perform joint disambiguation.

Lastly, the internal knowledge representation is translated into a Cypher query statement, then the query statement is executed, and the returned data is shown in tables and charts in the forms the user wants to obtain.

### The representation of clinical data and the form of supported questions

We choose main concepts, relations, and attributes in the common data model of the observational health data sciences and informatics (OHDSI) (http://www.ohdsi.org/web/wiki/doku.php?id=documentation:cdm) and propose a schema in graphical representations. With the schema, original data from CDR in relational database (RDB) is converted into a graph and stored in Neo4j.

**Fig. 2:**
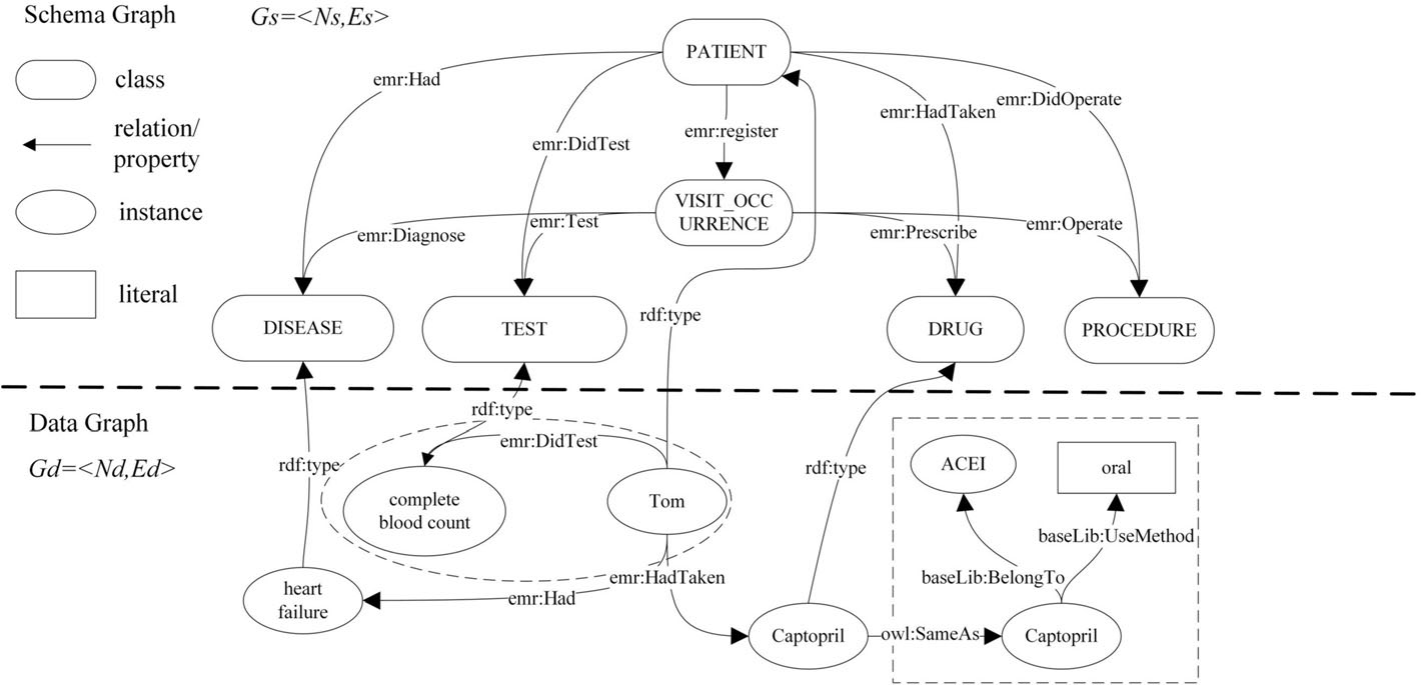
Patient graph connected with terminology graph. Patient graph contains schema graph and data graph. Patient-schema graph consists of concepts and relationships between concepts, and patient-data graph is the instantiation of the patient-schema graph, which consists of specific patients, diseases, drugs, etc

The OHDSI standard clinical database defines 12 tables. We choose frequently used tables in EMRs. Since we use an RDF-based knowledge graph to represent data, extending the schema to incorporate other concepts is very easy. The PERSON table stores demographic information about patients, such as gender and birthday. The VISIT_OCCURRENCE table stores the patient’s record of each visit, including the inpatient and outpatient. The PROCEDURE_OCCURRENCE table stores the surgery events that occur during the patient’s visit. The DRUG_EXPOSURE table stores the patient’s medication data. The DEVICE_EXPOSURE table stores the patient’s instrument data, such as pacemakers and bandages. The CONDITION_OCCURRENCE table stores the patient’s information about diseases and symptoms. The MEASUREMENT table stores the patient’s laboratory-examination information. We convert the tables into classes in Fig. 2, such as patient, disease, test, device, drug, procedure and visit_occurence.

The whole patient graph is denoted as graph G, which consists of the schema diagram Gs, the data graph Gd, and the relation R between Gs and Gd, namely G = < Gs, Gd, R >. In the schema diagram Gs = < Ns, Es >, Ns represents the set of class nodes in the graph, and Es represents the set of property edges. The edges correspond to the semantic relation among the classes. Similarly, in the data graph Gd = < Nd, Ed >, nodes set Nd contains the instance nodes and the literal nodes. An edge of the set Ed is connected by two nodes, and it represents a triple (subject, predicate, object). For example, in the center of data graph in Fig. 2, the node “Tom” has an edge connected to the node “complete blood count.” The edge “DidTest” indicates a relationship, whose subject is “Tom” and the object is “complete blood count.” The relationship R between the schema and the data graphs is represented by property rdf: type. Thus, R = {(instance, rdf: type, class)|instance ∈ Nd, class ∈ Ns}. The patient graph is connected with the clinical terminology graph [4], which collects major clinical terminologies in Chinese, and we have published the content on the Internet (https://old.datahub.io/dataset/symptoms-in-chinese). In this paper, we also extend the clinical terminology graph to add more drug-related information, such as drug types. The clinical terminology graph tends to be stable and does not change frequently, whereas the patient graph does. The entities in the patient-data graph are associated with entities in the terminology graph through the owl: sameAs or owl: equivalentClass relationships. We averaged three kinds of string distance to calculate owl: sameAs relationship, as shown in formula (1) in Concept linking. The optimal threshold is based on the Area Under Curve (AUC) of the manually annotated small-scale dataset.

We can propose more in depth analytic question after the patient graphs link with terminology graph. For example, after linking the ACEI drugs such as the captopril in the patient graph with the same term in the terminology graph, we can raise a query about the patients who have been treated with ACEI drugs. In Fig. 2, we use rdf as the name space for https://www.w3.org/1999/02/22-rdf-syntax-ns#, Schema for https://schema.org/, Owl for https://www.w3.org/TR/owl-features/, rdfs for https://www.w3.org/2000/01/rdf-schema#. For the patient graph and clinical terminology graph we designed, we use BaseLib and EMR as name spaces.

We used the Delphi method [5] to collect frequently used statistical questions with two rounds of questionnaires to the doctors. For the first run, we chose six doctors in our project from Shuguang Hospital of the cardiovascular disease department and cancer department. We talked with each doctor one by one in a room separately. We first let the doctor talk about their requirements at ease. After they finished, since not all the doctors were familiar with the statistical requirements, we also gave them one or two papers later and asked them whether the reports and figures in the paper were useful. We recorded all the requirements and presented them as natural-language questions. For the second round, we gave a website and asked the doctors to rank the questions. We chose the top 50 questions with the highest score.

The questions, whether they were from the cardiovascular disease department or the cancer department, are mostly similar. In general, doctors want demographic information, information on drug usage, etiological information that detect relations between causes and diseases, comparative effectiveness, and so on. Examples of demographic analysis may include patients with special diseases. The question “The patients who get diabetes and later suffer from heart failure” can be regarded as etiological analysis. The drug usages are most frequently asked, such as “What traditional Chinese medicine do the patients with coronary heart disease take?”. At present, our tool supports the following statistical types: lists, counts, aggregation (sum/averages), distributions, and ratios. The questions contain statistic vocabularies, such as “which”, “ratio” and “distribution”. Our tool also supports questions with logical operations (and/or/not), such as “What is the glucose test result for patients with hyperglycaemia and no hypertension who have taken hypoglycemic drugs?” These kinds of statistical questions with logical operations are frequently used in the comparative effectiveness occasions. The full list of question can be accessed from the GitHub URL we mentioned before.

**Fig. 3:**
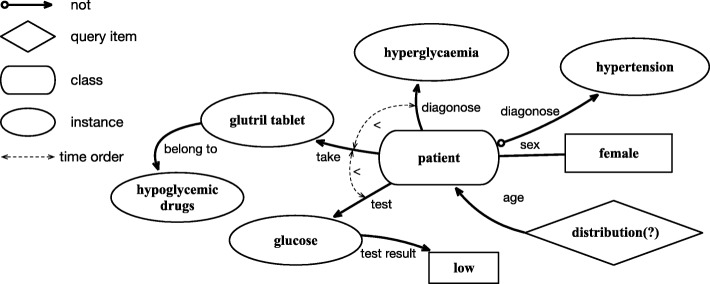
An example of the tree-like knowledge representation of questions

We represent the questions using a tree-like structure. A typical example of the structure is shown in Fig. 3. Nodes represent instances, classes, or attribute values, and edges represent relationships among these nodes. For the example, there are eight nodes and nine edges in “How many female patients with hyperglycemia and no hypertension have low levels of glucose tests after they took hypoglycemic drugs?” While the number of nodes and relations among clinical events give hints on the complexity of questions, we do not limit the length of the trees.

### Semantic parsing of questions

#### Word segmentation

Our tool uses the Chinese word-segmentation tool Jieba to segment the user’s question. However, if we use the out-of-the-box Jieba tool, the result of segmentation will be inaccurate because many medical terms are not included in the dictionary. For example, the tool segments a traditional Chinese medicine “复 方甘草口服溶液” (“compound glycyrrhiza oral solution”) into four parts: “复方,” “甘草,” “口服,” and “溶液.” A Chinese medical term dictionary is used to solve this problem. The dictionary is extracted from the medical knowledge graph we have constructed in our previous work [4] from medical-health websites and entity sets in EMR libraries. The knowledge graph contains commonly used symptoms, diseases, tests, and their relations in Chinese. To differentiate the patient graph constructed from EMR data, we will call the knowledge graph clinical terminology graph in this paper.

Time and periods are important concepts in medical care. To deal with different kinds of time-related expressions, such as “2015/6/10,” “2015年6月10日,” and “2015-6-10,” we define a series of rules to normalize time in a question into a format such as 20,150,610. We add a preprocessing step before the word segmentation.

We use two modes of Jieba. One is the accuracy mode, which returns the best answer. The other is the full mode, which returns all possible answers. The result of the accuracy mode may not be the right answer for our requirements. For example, “患有高血糖的女患者平均年龄是多少?” (“What is the average age of female patients suffering from hyperglycemia?”) The accurate mode of the segmentation result is “患有, 高血糖, 的, 女患者, 平均, 年龄, 是, 多少,? ”. However, “女患者” (“female patient”) is not an atomic concept that can be matched to the schema of the patient graph. In fact, the token contains two levels of information: “女” (“female”) is the value of the gender, and “患者” (“patient”) is the class patient. The correct segmentation result we need is “女/患者” (“female/patient”). Therefore, both the results of accurate and full modes are passed to the next phase. We will choose the best results later based on the grammar and patient-schema graph.

#### Concept linking

To analyze the internal composition of the question, we annotate 14 types of concepts to express it. They are Class, Instance, Property, Number, EnumValue, StatisticOperator, LogicalOperator, and so on, as shown in Table 1. Annotating numerical and time values by simple regular expression matching and using a small dictionary to annotate units, such as “mg/ml” and “mol/ml” are easy.

**Table 1:** Types of concepts used in concept linking step

Concept type	Example	Concept type	Example
Relation	患有 (suffer from)	RangeSeparator	–
Instance	心衰 (heart failure)	TimeOperator	先 (before)
Class	疾病 (disease)	LogicalOperator	和 (and)
Property	年龄 (age)	StatisticOperator	比例 (ratio)
Number	30.96	ArithmeticOperator	等于 (equal)
EnumValue	女 (female)	Unit	天 (day)
TimeValue	[20170909]	Not	没有 (no)

The challenges arise from linking unknown tokens to classes, instances, relations, and properties. For example, the test item “blood glucose” is often shortened as “glucose”. Therefore, we use similarity matching to link the token to its possible concepts.

Link token to concept types based on string similarity. Let E be the set of names of class, entity, relation, attribute, and enumerated attribute value. S(ej) is the set of ej with its synonyms, ej ∈ E. For the input tokeni and the candidate ej, the similarity among all the values in tokeni and S(ej) is calculated, and the highest similarity score is used as the score of the candidate ej. In the end, the top k names are selected from E as the candidate corresponding to tokeni in the query.

The similarity function σ considers the Levenshtein distance, trigram, and longest common substring, as shown in formula (1):

1 $$ \upsigma \left({token}_i,{e}_h\right)=\frac{L\left({token}_i,{e}_h\right)+T\left({token}_i,{e}_h\right)+ LCS\left({token}_i,{e}_h\right)}{3} $$

in which,

2 $$ L\left({token}_i,{e}_h\right)=1-\frac{Levenshteindistance}{\max \left(\left|{token}_i\right|,\left|{e}_h\right|\right)} $$

3 $$ T\left({token}_i,{e}_h\right)= Jaccard\left( trigram\left({token}_i\right), trigram\left({e}_h\right)\right) $$

4 $$ L\mathrm{C}S\left({token}_i,{e}_h\right)=\frac{LongestCommonSubstring}{\max \left(\left|{token}_i\right|,\left|{e}_h\right|\right)} $$

For example, on the token “glucose”, we get the two top candidate words, “blood glucose” and “glucose oral liquid”. While both the words belong to the instance set, they are of different types. Therefore, while the concept-type sequence is “Relation Instance Not Relation Instance Class Relation Instance TimeOperator Instance Property EnumValue Property EnumValue Class StatisticOperator,” the instance links to multiple candidate words, resulting in ambiguities. We will both save these two candidates, and delay the disambiguation task to a later section.

Supplement missing attribute. Utterances in natural language often omit the words about attribute names. For example, the attribute name “age” in the question “How many female patients over 60 years old have hyperglycaemia?” is omitted; only the attribute value “60 years old” appears. Therefore, we use the schema information to supplement the missing attributes since they can cause grammar parsing in the later step to fail. For the previous example, the unit “years old” corresponds to the attribute “age” in our schema graph, and “female” is an enumeration value of attribute “sex”.

#### Grammar parsing

##### Context-free grammar

We use the context-free grammar G = [VT, VN, P, S] to describe the clinical research questions. VT is the terminator set; the elements in the set are concept types listed in Table 1. Each terminator represents the basic units that make up the sentence. VN is a set of nonterminating set, VN = [Condition, RelationCondition, PropertyCondition, ...]. Each non-terminating element represents a chuck of information. P is the set of production rules. Table 2 lists the generative form of the grammar, and S is the beginning of the grammar, representing the whole sentence.

**Table 2:** Grammar definition with production rules

1	S - > QueryItem | QueryItem S | Condition S | Condition ‘Class’ S
2	Condition - > RelationCondition | PropertyCondition
3	Condition - > Condition ‘LogicalOperator’ Condition
4	Condition - > RelationCondition ‘TimeOperator’ Condition
5	RelationCondition - > ‘Relation’ ‘Instance’ | ‘Not’ ‘Relation’ ‘Instance’ | TimeBlock ‘Relation’ ‘Instance’ | TimeBlock ‘Not’ ‘Relation’ ‘Instance’
6	TimeBlock - > ‘TimeValue’ | ‘TimeValue’ ‘LogicalOperator’ | ‘TimeValue’ ‘RangeSeparator’ ‘TimeValue’
7	PropertyCondition - > ‘Property’ PropertyValue | ‘Instance’ ‘Property’ PropertyValue
8	PropertyValue - > ‘EnumValue’ | NumericValue | NumericRange | TimeBlock
9	NumericValue - > ‘Number’ | ‘Number’ ‘Unit’
10	NumericRange - > NumericValue ‘RangeSeparator’ NumericValue | ‘ArithmeticOperator’ NumericValue
11	QueryItem - > ‘Property’ ‘StatisticOperator’ | ‘StatisticOperator’ ‘Property’ | ‘Relation’ ‘StatisticOperator’ ‘Class’ | ‘Instance’ ‘Property’ ‘StatisticOperator’

As shown in Table 2, the questions are composed of one or more semantic-related chunks. These include property-condition, relation-condition, time-block, and query-item chunks. Relation-condition chunks can consist of relationships and instances, such as “患有高血糖” (“suffering from hyperglycemia”), in which “患有” (“suffering from”) indicates a relationship and “高血糖” (“hyperglycemia”) represents an instance. There is a hidden concept “患者” (“patient”) in the relationship. However, users usually put it in the end of the condition when multiple condition exists. It can also be negative relation-condition chunks, such as “没有患有高血糖” (“not suffering from hypertension”).

Property-condition chunks can be composed of class/instance, attribute names, and attribute values, including numeric, enumerated, and date attributes. For example, “葡萄糖化验结果偏低” (“glucose test results are low”), in which the “葡萄糖”(“glucose”) is an instance, “化验结果” (“test results”) belongs to attributes, and “偏低” (“low”) belongs to enumeration values. The subject can also be omitted, for example, “住院天数超过10天” (“the day of stay in hospital exceeds 10 days”), where “住院天数” (“the day of stay in hospital”) is an attribute and “超过10天” (“exceeds 10 days”) indicates numeric values of attributes in a range form.

The query-item chunks represent the statistical operations on the records that satisfy the combination condition. For example, the question about etiology “What is the ratio of heart failure in patients with hypertension, diabetes, and coronary heart disease?” uses the ratio operation. The question about effectiveness “How many female patients with hyperglycemia and no hypertension have low levels of glucose tests after they took hypoglycemic drugs?” contains several logic and time operators to filter the patient’s records and uses the count operation.

All the previous examples show the expressiveness of our grammar, and these kinds of questions are complex and are hard to interpret in current fact-oriented knowledge-based QA tools.

##### Using Stanford parser as complement

Although context-free grammar covers most questions used in clinical research, grammars still fail to parse in some special cases. For example, “patients who suffer from hyperglycemia and hypertension”, our grammar cannot capture this relationship because of the remote dependency of “suffer from” and “hypertension.” So, we use Stanford parser to do a dependency analysis of the sentence, which can obtain the parallel relationship “conj” between “hyperglycemia” and “hypertension.” Through the parallel relationship, we get the verb “suffer from” of “hypertension.” The concept-type sequence is extended to “Relation Instance LogicalOperator Relation Instance” for normal parsing. Currently, we use “cc” and “conj” relations, to solve the above problem. In the future, we will use more parsing results of Stanford parser to make the input query more flexible.

#### Semantic representation with disambiguation

We convert the sequence of concept types and the results of parse tree into an internal knowledge representation. As mentioned in Concept linking, the representation is basically triples connected with logical and time operators, coupled with distribution operators.

The relation condition chunk typically consists of two tokens, such as <Relation, Instance>. To convert the chunk into a triple, we may use the definitions of domain and range in the patient-schema graph the nearest class node to find the subject of the triple. For instance, in “eat Coldrine”, “eat” is binded with the predicate “take” since the schema graph contains a synonym relation < eat, sameAs, take >. In the concept linking step, “Coldrine” can be linked to “cold” and “Coldrine capsule”. Based on the patient-schema graph, < patient, suffer from, cold >、 < patient, take, Coldrine capsule >, the latter is chosen since the predicate is “take”. The property-conditional chunks are similar to relation-condition chunks. Our query combines statistic operators with classes and property values. With the help of query-item chunks, we can find out the object that the statistic operator works on.

Among the Not, LogicalOperator and TimeOperator that appear in the grammar tree, Not has the highest priority. TimeOperator is least prioritized among the three. We use “()” to indicate priority. Besides, the chronology of the relations may not explicitly appear in the sentence. We use some heuristic rules to detect them, which can be a part of the patient-schema graph.

About the example in Overview of the method, the ultimate expression is (<patient, suffer, hyperglycemia> (not <patient, suffer, hypertension>)) after <patient, take, hypoglycemic drugs> after (<patient, have test, blood glucose> < blood glucose, test result, low>) < patient, sex, female> < patient, count,? > .

#### Cypher translation

**Fig. 4:**
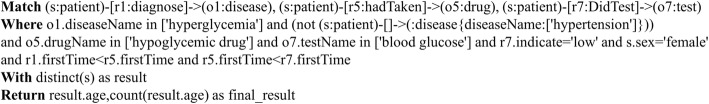
Cypher query example

This step is to translate the semantic representation of the query into Cypher, which is a query language of graph database Neo4j. The translation process consists of three parts: the translation of match clause, where clause, and return clause. Similar to the tree-like semantic representation, the match clause is also made up of nodes and edges, in which “()” denotes node and “-[]- >” denotes edge. The attribute-value constraint in semantic representation corresponded to the conditions in the where clause. The target attribute values or classes in the semantic representation corresponds to the return clause in the query statement. For example, the patient node directly connects with the disease node, the test node, and the drug node; and the structure corresponds to the match clause in Fig. 4. The gender, disease, and drug constraints of the where clause in Fig. 4 correspond to the attribute constraints of class-node patients, whereas the time constraint between “take” and “test” maps from TimeOperator in the graph. The return clause ultimately returns the number of patients who satisfy the subgraph.

**Fig. 5:**
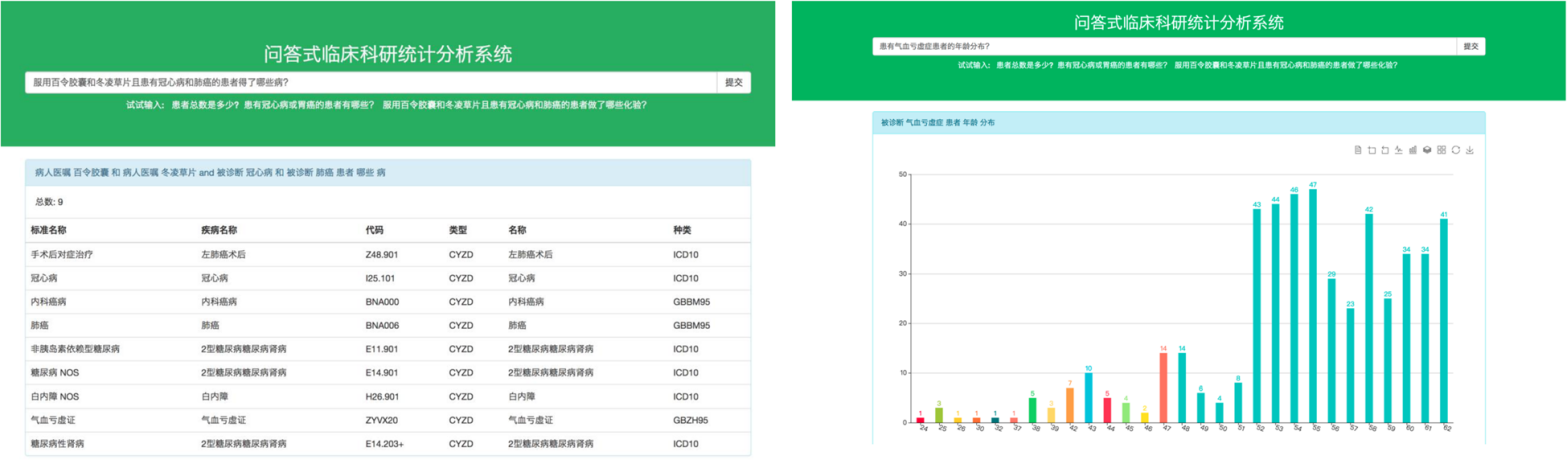
User interface of our tool. The statistics type of the left figure is list, and the right figure is distribution

We execute the Cypher query in Fig. 4. The results are returned with the statistics type to the front end. Depending on the result-data type and the statistics type, the front end loads different diagrams. For example, if the statistics type is a list, a table is loaded. If the type is a distribution, the bar chart is loaded. The results are shown in Fig. 5.

## Results

### Experimental setup and benchmark

Since no standard question sets are applicable in our contexts for evaluation, we collect our questions from both related literature and clinical doctors. We search literature on Wanfang Data, a literature-resource library with major research journals in Chinese, using different combinations of three groups of key words: electronic medical records, electronic health records, statistical, data analysis and epidemiological investigation, clinical research. Out of the 50 returned papers, 16 are with statistical problems in clinical research. For English questions, we also select published papers from 2016 to 2017 in the CALIBER research project. About 12 papers with statistic requirements (statistical information is presented in a tabular form in these English literature) from EMR are selected. There are altogether 28 papers in Chinese and English, and we gathered 161 questions from them. Since a part of the question set is taken from CALIBER, and is translated by the doctor. While translating these questions into Chinese, many entities do not exist in the current system knowledge base, such as “HDL cholesterol ratio,” “England,” and “depression.” We replace them with similar entities in our knowledge base. For example, “How many female patients in England” will be replaced with “How many female patients in Shanghai?”. We merge these questions with 50 others collected from the doctors and get 211 questions altogether. The question lists can be accessed from our GitHub URL.

Besides, we organize a joint team with physicians and IT professionals to generate the gold standard (benchmark results) for the 211 questions. The team are divided into three groups. Each group contain two persons, one is a physician whose task is to verify whether the query is correctly interpreted, the other is an IT professional who execute the query against the dataset, then both of them verify whether the result is correct. Each question is assigned to two groups. If the two groups get different answers, then the third groups decide the ultimate results.

The EMR data used to test the performance and accuracy of our tool in this paper is from Shanghai Shuguang Hospital, which is a large hospital for integrated Chinese and Western Medicine. It has used EMR systems for more than a decade. Patient health-care encounters are well-documented and time-stamped. The hospital has built their CDR for three years. We construct a sub-repository with congestive heart failure for clinical research. There are 486,525 edges and 21,728 vertices in our graph, and the average degree is 44.78. As shown in Table 3, there are 383 test items, 1156 drug items, 324 disease items, and 49 procedure items in the terminology library, a total of 6035 patients in the heart-failure repository resulting in 13,781 edges between patients and hospitals, 112,838 edges between patient and tests, 324,818 edges between patients and drugs, 16,241 edges between patient and diseases, and 18,847 edges between patients and procedures.

**Table 3:** Data size in performance evaluation

Node	Quantity	Edge	Quantity
Hospitalization	13,781	InHospital	13,781
Test	383	DidTest	112,838
Drug	1156	HadTaken	324,818
Disease	324	Diagnose	16,241
Procedure	49	DidOperate	18,847
Patient	6035		

We define coverage and precision as the quality indicators of the tool. Here, coverage means the percentage of natural language questions that our tool can recognize and return results. Precision means for all the results returned from our tool, the percentage of correct results is returned. Coverage is more suitable to use than other similar ones, such as recall, since we can extend our tool with more rules on grammar or translation, and the tool can cover more types of questions.

5 $$ \mathrm{coverage}=\frac{The\ number\ of\ questions\ that\  our\  system\  can\  return\ result\mathrm{s}}{The\ number\ of\ questions\ } $$

6 $$ \mathrm{precision}=\frac{The\ number\ of\ questions\ that\  our\  system\  can\  output\ the\ correct\ results}{The\ number\ of\ questions\ that\  our\  system\  can\  return\ results\ } $$

### Experimental evaluation on the coverage and accuracy of the tool

The evaluation result is shown in Table 4. Out of the 211 questions, results for 165 questions are returned. The overall coverage rate is 78.20%. We also classify the questions according to the number of nodes in the question. We can see that most questions contain two or three nodes. However, the coverage and precision has no direct relation with the number of nodes.

**Table 4:** The result of coverage and precision

Number of Nodes	Number of Question	Coverage (%)	Precision (%)
1	20	95.00	100.00
2	112	75.00	98.81
3	62	74.19	95.65
> 3	17	94.11	81.25
total	211	78.20	96.36

The remaining 46 questions whose results are not returned can be divided into four categories:

1. There is one question with a statistical vocabulary “annual growth rate” that is not supported by the tool. We can solve this problem by adding this word to the statistical vocabulary and setting up its related function
2. There are 17 questions whose semantics cannot be interpreted directly, for example, “What is the comparison of the prevalence of hypertension between 1991 and 2014?” Currently, the tool’s grammar cannot parse and interpret the term “comparison” correctly. However, we can extend the vocabulary and parse them into two tree-like structures later.
3. Two questions contain operations not supported in Neo4j, for example “What is the sex ratio of the top 10 diseases among inpatients in 2012?” we need to sort the group by operation for each disease first, but it is not supported in Neo4j.
4. Twenty-six questions contain properties that do not appear in our patient graph, such as “current smoker.” as mentioned before, adding new nodes and edges to extend our patient graph is easy
  Results for six questions are wrong, and the precision is as high as 96.36%. The reasons are the following:
5. Questions, which collected from doctor, contain vocabularies that do not exist in our knowledge base. For example, “How many patients are suffering from sepsis, heart failure, and coronary heart disease?”, “sepsis” is not in our knowledge base. Our tool automatically ignores the entities, so the result is the same as “How many patients are suffering from heart failure and coronary heart disease?” To solve the problem, we plan to add a named entity recognition step. If the entity cannot link to any entity in the knowledge base, we will prompt the user.
6. Entities in the question are linked to wrong entities in the knowledge base. Entities in the question may not exist in the knowledge base, so they are linked to the wrong entities, for example, “Who are the patients with coronary heart disease and rheumatic heart disease?” The tool will link rheumatic heart disease to heart disease. In this case, a larger knowledge base is required.
7. Our tool can only recognize the time relation between two entities where temporal preposition exists. In “High blood pressure patients with coronary heart disease after heart failure”, our tool can only know that heart failure occurs before coronary heart disease, but the hidden semantics that hypertension happens earlier than the previous two cannot be captured. For this case, we intend to define a set of heuristic rules to recognize such domain-specific implications.

If none of the above three situations occur in the question, the accuracy will be 100%. At the same time, we are trying to prevent errors that may be caused by the above situations. So, we output the results of the parser to the doctor for review.

### Tool performance evaluation

The test environment is a PC with Centos 6.8, 16G memory, and 4 CPU core. The JVM maximum heap size of Neo4j is set to 4G. Fifty questions are used; the result is shown in Table 5. The query time of 78% questions is within 1 s. These kinds of questions mostly contain less than three nodes and has no logical operation OR. There are three cases which return time is more than one second:

1. The number of nodes in questions is large and mostly more than three, and the query is generally completed within two seconds. These questions accounted for 10% of the question set.
2. Not only is the number of nodes more than three, but it also contains several logic operators, especially OR and TimeOperator. These kinds of questions require two to six seconds, and account 2% of the question set.
3. The start node of the Cypher query is a large collection, which makes it time-consuming to filter and match time operations later. The query time may take as long as 25.88 s. We encounter five questions with the problem. In general, the performance is basically acceptable in current phase.

**Table 5:** The results of tool performance evaluation

Time	Number	Typical types	Example
t < 1	39	Less than three nodes and no logical operation OR	2013年10月入院的患者(Patients admitted to hospital in October 2013)
1 s ≤ t ≤ 2 s	5	More than three nodes	患有冠心病没吃中药的患者有哪些(Who are the patients with coronary heart disease and taking no Chinese medicine)
2 s < t ≤ 6 s	1	More than three nodes and contain several logic operators, especially OR and TimeOperator	2013年之后吃了至灵胶囊或用了白玉膏或用了艾迪注射液的男性肺癌患者的年龄分布(The age distribution of male patients with lung cancer who took the ZhiLing capsule or BaiYu cream or injected Addie after 2013)
6 s < t	5	Do filtering and matching time operations	住院次数大于4次的肺癌患者做了哪些手术(What procedures did lung cancer patients with more than 4 hospitalized records take?)

## Related work

Our work is closely related to two important research topics: question answering using a knowledge base (KB-based QA) and question answering on statistical linked data.

The traditional methods for KB-based QA is based on semantic parsing [6–13]. It resolves the natural-language question into a logical representation expressing the semantics of the question, and then the logical expressions are translated into structured queries. Answers are found with queries executed on the knowledge base. There are many challenges during the process. In particular, [12] uses an integer-linear program to solve several disambiguation tasks jointly in this pipelined process, including phrase segmentation, phrases, phrases grounding, and the construction of SPARQL triple patterns. To represent the natural-language question, [13] proposes a semantic query graph, and RDF QA problem is reduced to a subgraph-matching problem. In this way, they solve the disambiguation problem that traditionally has the exponential search space and results in slow responses. To find logical predicates for a given question on Freebase, [7] uses machine learning, and [6] deals with the situation when a structurally simple question is mapped to a k − ary relation in the knowledge base.

The overall performance of the semantic-parsing method is not promising since errors may occur in each step from converting original questions to logic forms. Another way for QA on a large knowledge base is to convert both questions and answers to similar representations and check similarities between the two representations. Also, [14] transforms original questions into feature graph, and the relevant nodes in Freebase into topic graphs, and then they extract and combine features from the two graphs and treat QA on Freebase as a binary classification task. With the prevalence of word-embedding learning, the knowledge-representation tasks become much easier. The representations of questions and corresponding answers can be similar to the embedding space with vector embedding of words and KB triples, which forms the basis of deep learning-based method of QA [15–18]. While earlier work [15, 17] simply encode questions as a bag of words, more recent work utilizes more structured information. For example, [16] relies on multicolumn convolutional neural networks to represent questions from three different aspects: answer path, answer context, and answer type.

Both QALD and TREC have special for medical field. Recently, the TREC-CDS (clinical decision support) track requires to retrieve relevant scientific articles that contain the answers to medical questions. Goodwin et al. [19] propose a two-step approach. They discovered the answers by utilizing a probabilistic medical knowledge graph that is constructed from many EMRs. Then they selected and ranked scientific articles that contain the answer. TREC-CDS focuses more on retrieving texts than Q&A. Liu Fang et al. [20] used template-based methods to implement the medical questions and answers in Chinese. They define 300 templates in medical field. However, the paper does not address any challenges in this process (e.g. templates conflicts or terminology segmentations).

QALD 2016 has a special task about question answering on statistical linked data [21]. The application context to give answers to statistical questions is quite similar to ours. However, their queries are fixed on the dataset of cube-based multidimensional RDF data. The cube uses notions in OLAP model, such as dimensions, measures, and attributes, which are introduced in [22]. CubeQA algorithm designed in [21] uses a template-based pipeline approach similar to [14] and achieves a global F1 score of 0.43 on the QALD6T3-test benchmark. Faceted search can be regarded as an alternative approach to QA. Faceted search requires users to interact with the system, and users select different facets to filter the dataset. Such systems include Ontogator [23], Slash-Facet [24], BrowseRDF [25], gFacet [26], VisiNav [27], and SemFacet [28]. Cubix [7] and Linked Data Query Wizard [29] also support OLAP model.

The cube-based approach requires the original graph-based dataset to be converted into multidimensional data. It not only needs a large amount of skills and hard work but also limits the possible ways questions are raised. Faceted-search approach, in general, does not support complex logical operations, such as negative, or relations, between events, which is important in the medical field.

## Conclusions

We designed and implemented a statistical analysis tool in the natural language, which aims to help doctors who lack IT skills and ease their clinical research. Our tool shows great flexibility in processing different kinds of statistic questions, which is important in domain-oriented applications and is also the key difference between our tool and the current fact-oriented knowledge-based QA tool. Besides, our designed clinical knowledge graph is easy to extend and to connect with other knowledge graphs.

In the future, we will use more dependency relation in Stanford parser to allow questions to be expressed more flexibly. Once enough user logs are accumulated, we plan use machine learning techniques to handle these question-translation challenges. Since, the schema of our patient graph and our questions representation do not limit our questions to statistic ones, we plan to combine statistical requirements with normal KB-based QA requirements in our tool.

## Abbreviations

ACEI: Angiotension converting enzyme inhibitors
CDR: Clinical data repository
CSV: Comma-separated values
EMR: Electronic medical record
OHDSI: Observational health data sciences and informatics
QA: Question and answering
QALD: Question answering over linked data
RDB: Relational database
SQL: Structured query language
